# The Emerging Role of TXNIP in Ischemic and Cardiovascular Diseases; A Novel Marker and Therapeutic Target

**DOI:** 10.3390/ijms22041693

**Published:** 2021-02-08

**Authors:** Alison Domingues, Julia Jolibois, Perrine Marquet de Rougé, Valérie Nivet-Antoine

**Affiliations:** 1 INSERM 1140, Innovative Therapies in Haemostasis, Faculty of Pharmacy, Université de Paris, 75006 Paris, France; alison.domingues@unimi.it (A.D.); juliajolibois@live.com (J.J.); perrine.marquet@gmail.com (P.M.d.R.); 2 Clinical Biochemistry Department, Assistance Publique des Hôpitaux de Paris, Necker Hospital, 75015 Paris, France

**Keywords:** TXNIP, cardiovascular diseases marker, oxidative stress, metabolic disorders, post-ischemic revascularization

## Abstract

Thioredoxin interacting protein (TXNIP) is a metabolism- oxidative- and inflammation-related marker induced in cardiovascular diseases and is believed to represent a possible link between metabolism and cellular redox status. TXNIP is a potential biomarker in cardiovascular and ischemic diseases but also a novel identified target for preventive and curative medicine. The goal of this review is to focus on the novelties concerning TXNIP. After an overview in TXNIP involvement in oxidative stress, inflammation and metabolism, the remainder of this review presents the clues used to define TXNIP as a new marker at the genetic, blood, or ischemic site level in the context of cardiovascular and ischemic diseases.

## 1. Introduction

Cardiovascular diseases remain a major cause of death worldwide and are increasing due to the ageing population and poor eating habits. The pathological changes are originally characterized by metabolic disorders and endothelial dysfunction. Oxidative stress plays an important role and induces vascular-related gene expression, promoting local inflammatory response and cell life and death dysregulation. When oxidative stress occurs, vascular walls produce excessive reactive oxygen species (ROS), which causes damage to the structure and function of endothelial cells. That enhances the inflammatory response of the vascular wall and impairs vascular function or revascularization. ROS are produced continuously during cell metabolism and are used as mediators in many biological processes. Specifically, ROS reversibly activate signaling pathways that trigger adaptation systems in the cell. Previous works have associated excessive ROS with age-related pathologies [1,2,3,4,5,6,7]. However, recent reviews still report that excessive ROS can lead to diseases and pathological conditions [8,9,10,11]. Thioredoxin interacting protein (TXNIP) is a metabolism- oxidative- and inflammation-related marker induced in cardiovascular pathologies and could represent an emergent link between physiopathology and cardiovascular events. More precisely, TXNIP has been widely described as a pro-oxidant compound [12,13], but it is also a regulator of metabolism [14,15,16,17], a modulator of the inflammatory [18,19] or angiogenic response [20,21], and an antiproliferative and pro-apoptotic agent [22,23]. Clinically, genetic association studies have shown that polymorphisms affecting TXNIP expression are linked to hypertension and arterial stiffness and increase the risk of coronary heart disease [24,25,26]. Epigenetic modifications of TXNIP are also associated with risks of cardiovascular diseases [27,28]. Additionally, blood or mononuclear blood cells’ mRNA TXNIP levels have been related to coronary and heart diseases [29,30,31].

Finally, in this review, we propose an overview of half a decade of work analyzing TXNIP as a marker of cardiovascular risk and diseases. The mechanisms involved are specified and TXNIP is identified as a potential target for preventive and curative medicine in cardiovascular and ischemic diseases.

## 2. TXNIP is a Multifunctional Protein

TXNIP is a 46-kDa ubiquitously expressed protein that contains 391 amino acid residues and is encoded on chromosome 1q21.1. TXNIP is an α-arrestin protein that regulates pleiotropic biological responses [32,33,34]. TXNIP appears to perform certain functions through multiple binding partners [35], which are summarized in Table 1.

### 2.1. TXNIP is a Protein Shuttle

The structure of TXNIP and the presence of “arrestin-like” domains suggest the ability of this protein to interact with other proteins but also to participate in cell trafficking and particularly in the transport of proteins with which it interacts. TXNIP is almost exclusively localized in the nucleus in healthy cells [36,133]. Nevertheless, TXNIP is able to translocate into the mitochondria under fructose stimulation, into the cytosol, and to the membrane in response to physiological levels of ROS [134]. During its translocation, it also carries several proteins. Thioredoxin (TRX), a major antioxidant enzyme, is carried by TXNIP to the membrane [36]. This interaction is central to the regulation of the TRX-TXNIP system. In addition to its role with TRX, TXNIP can also interact with HIF-1α. TXNIP leads HIF1- α outside the nucleus to the cytosol where the factor will be degraded [38]. TXNIP is also responsible for the nuclear translocation of NF-κB [39,40]. In parallel, TXNIP is degraded by the ubiquitin-proteasome using the protein ITCH. Interestingly, the regulation of TXNIP lifetime appears to be crucial for the control of TRX oxidoreductase activity [41]. Finally, the overexpression of ITCH in cardiomyocytes is reported to ameliorate reactive oxygen species-induced cardiotoxicity through the thioredoxin system [42]. In this case, TXNIP degradation is driven by ITCH binding to the “arrestin-like” domains [43].

### 2.2. TXNIP in the TRX-TXNIP System

This system is highly conserved in almost all species, from bacteria to higher eukaryotes [135,136]. By virtue of its location, TRX is a protein of choice for fighting oxidative stress in the vessel. It exerts antioxidant activity by allowing the reduction of target proteins via the formation of disulfide bonds between two cysteine residues of its active site (Cys-Gly-Pro-Cys). This results in the oxidation of TRX and the reduction of residues on target proteins [32,44]. Then, TRX can be reduced by a flavoprotein oxidoreductase, called thioredoxin reductase (TrxR), which uses the NADPH as a co-factor, allowing it to be able to exert its antioxidant role [45]. TRX plays an important role in maintaining redox balance and cell signaling by participating in sulfhydryl reactions (e.g., reducing cysteine residues) and by interacting with various components of signaling pathways [46]. Several studies have shown that TRX activity could be modulated by a negative endogenous regulator: TXNIP [47,48]. TXNIP interacts with the catalytic center of reduced TRX and inhibits its reducing activity [44,48,49]. TXNIP deletion results in decreased ROS in vascular smooth muscle cells (VSMC), for example, and increases the antioxidant potential of TRX in vitro [135]. TXNIP is described as a pro-oxidant compound, but its role does not end with a simple modulation of the redox balance; it also acts as a transporter of TRX [37].

### 2.3. TXNIP and Its Role in Oxidative Stress

Oxidative stress is recognized as the first step in endothelial dysfunction, leading to vascular damage [2,50] and impaired revascularization [21]. The decreased expression of TRX, associated with increased expression of TXNIP, is implicated in endothelial dysfunction associated with aging and, in this context, increased expression of NADPH oxidase is also reported [51]. More interestingly, reduced endothelial expression of TXNIP is associated with an increased TRX and decreased NADPH oxidase expression protecting the endothelium from dysfunction induced by metabolic disorders [13,52]. Overexpression of TXNIP in vitro is associated with oxidative stress induced by mitochondrial ROS or NADPH oxidase [56]. Furthermore, given the role of TXNIP in inhibiting the antioxidant activity of TRX, its role in oxidative stress seems obvious [40,59,63,65,137,138]. Blocking its expression then induces a decrease in oxidative stress [139]. An in vitro study shows that TXNIP promotes nitrosative stress via its inhibitory action on TRX and repressing TXNIP, and thereby facilitates thioredoxin-mediated denitrosylation [140]. Interestingly, pharmacological inhibition of TXNIP in an in vivo model of ischemia-reperfusion has been associated with increased TRX activity [53]. A study in a mouse model of diabetes also shows the excess of ROS and the establishment of oxidative stress resulting from the decrease in the activity of TRX due to its inhibition by TXNIP [54]. Indeed, when glucose is high, TXNIP mediates the production of ROS via mitochondria and NADPH oxidase [56,57]. The resulting oxidative stress arises from overexpressed TXNIP, leading to endothelial dysfunction and impaired vasorelaxation [12,21].

### 2.4. TXNIP: Link between Oxidative Stress and Inflammation

**Figure 1:**
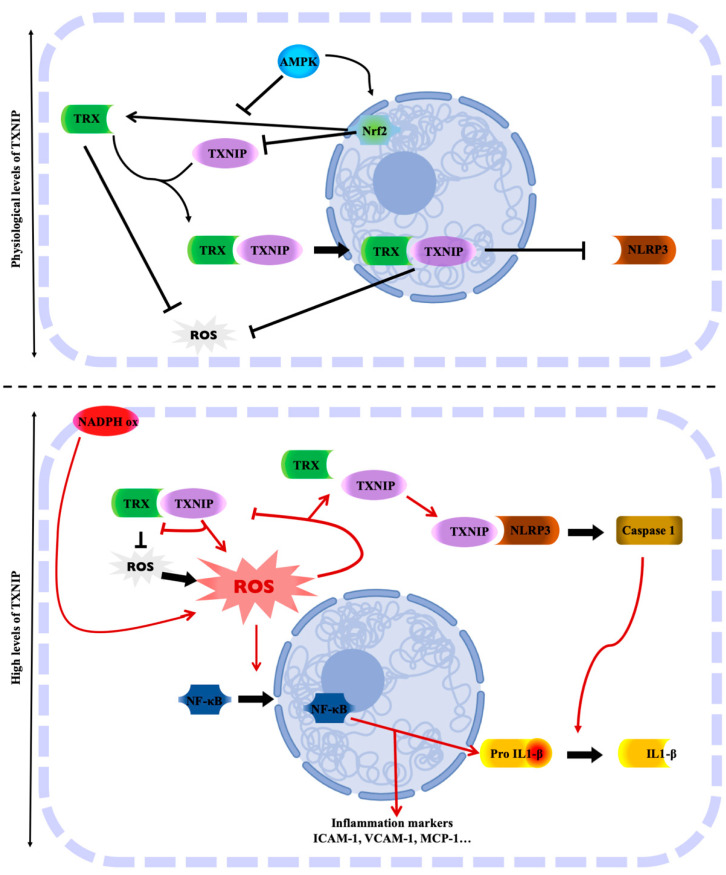
TXNIP is the molecular link between the regulation of oxidative stress and inflammation. Physiological levels of TXNIP balance the regulation of TRX oxidative stress. TRX is free from TXNIP, whose level is controlled by several factors. However, high levels of TXNIP inhibit TRX activity, resulting in oxidative stress with the accumulation of ROS. The oxidative stress status allows TXNIP to activate the NLRP3 inflammasome and trigger cell inflammation. Created with BioRender.com.

TRX is a protein which, in addition to its role as an antioxidant, has anti-inflammatory properties. It is therefore a crucial protein in the protection of age-related vascular damage. Many articles suggest that TRX is an anti-inflammatory molecule at both intracellular and extracellular levels [141,142]. Conversely, TXNIP has a pro-inflammatory role leading to cardiac, vascular and endothelial dysfunctions [13,19,21,66,67,68,143,144]. Numerous studies conducted since the 2000s suggest that TXNIP may bind to the NLRP3 inflammasome, which enhances the inflammatory response, as reviewed almost a decade ago [65,69,70,71,72,73,74,75,76,77,78,79,80,81,82] and summarized in Figure 1. TXNIP and NLRP3 physically interact to activate the inflammasome [83]. The inflammasome is the multiprotein complex that controls the activation of caspase-1 in the innate immune system. Then, it causes the maturation of IL-1β. ROS are the major activators of the NLRP3 inflammasome. The physical interaction between TXNIP and NLRP3 could explain the activation of the inflammasome in a ROS-sensitive manner [18,84,85]. Zhou et al. suggested that under unstressed conditions, TXNIP is bound to TRX, and the NLRP-3 inflammasome is inactive due to a lack of interaction between TXNIP and NLRP3. However, under oxidative stress conditions, the generation of ROS facilitates TRX-TXNIP dissociation, thereby increasing NLRP3-TXNIP interaction [84] (Figure 1). These observations were then demonstrated in podocytes [86] or in response to LPS [87]. More recently, in adult mice with metabolic disorders, ROS accumulation results from endothelial dysfunction with decreased TRX and increased NADPH oxidase endothelial expression, leading to oxidative stress and NLRP3 inflammasome activation in the aortic wall [13,88]. In addition to the oxidative stress, TXNIP upregulation through the p38-FOXO1 axis seems critical for NLRP3 activation [89]. Endoplasmic reticulum stress is also associated with TXNIP-NLRP3 activation in the context of endothelial dysfunction [90]. Interestingly, the deletion of endothelial TXNIP in mice or in vivo anti-TXNIP treatment protects from oxidative stress and NLRP3 inflammasome activation [13,21]. Metformin or other compounds are also used to lower TXNIP aortic levels in vivo or endothelial levels in vitro in order to restrain NLRP3 activation and protect from endothelial dysfunction and cardiovascular risk factors [60,67,87,91,92,93,94,95,96,97,98,99,100,101]. The regulation of the NLRP3 inflammasome by the TRX-TXNIP complex is believed to be controlled by Nrf2 and AMPK [60,102,103,104,106,107]. The overexpression of TXNIP activates the TLR4-NF-κB-NLRP3 inflammasome signaling pathway with increased MyD88, NLPR3 inflammasome, and ASC expression, as well as the increased phosphorylation of lκBα and p65, thus promoting downstream NF-κB activation [109]. The blunted inflammatory response is associated with a decrease in NF-κB nuclear translocation. In fact, in hyperglycemic conditions, the overexpression of TXNIP leads to an increased expression of inflammation genes via chromatin modifications and by promoting nuclear translocation of NF-κB [39]. In addition, TXNIP also promotes inflammation in the endothelium in response to disturbed flow [111]. The expression of inflammatory markers and adhesion molecules such as ICAM-1, VCAM-1, and MCP-1 are diminished in VSMCs from TXNIP knockout mice [110]. This inflammation state can trigger cell senescence or cell death characterized as pyroptosis, which is hampered with the decrease of TXNIP [66,78,92,93,105]. The ROS-TXNIP-NLRP3 pathway can actually be enabled for a long time via an IL-1β-mediated positive feedback loop [145].

### 2.5. TXNIP and Its Role in Metabolism

TXNIP has also attracted considerable attention due to its wide-ranging functions impacting several aspects of energy metabolism, as already reviewed elsewhere [14,15,16,146,147]. TXNIP is known to modulate cellular glucose utilization, the mitochondrial oxidation of metabolic fuels, and fasting-feeding transition [112,148]. TXNIP is implicated in adaptation to acidosis and, interestingly, it is associated with ATP generation [114]. Consequently, TXNIP appears to be an important regulator of glucose homeostasis via the regulation of gluconeogenesis in the liver [149]. In addition to metabolic disturbances, the total deletion of TXNIP induces the development of hemorrhages and hepatic steatosis which can lead to death [150]. Nevertheless, a recent genetic study in a family with homozygous nonsense mutations shows that suppression of TXNIP expression is non-lethal in humans [151]. A number of studies have therefore shown that TXNIP has a role to play in metabolic control, partially independent of its ability to bind to TRX [115,152]. Insulin and cellular glucose influx reciprocally regulate TXNIP expression in humans: glucose influx positively regulates TXNIP expression and its suppression by insulin [115,153]. These results are consistent with a recent study showing that IGF-1 could suppress TXNIP expression [116]. In addition, studies have shown that TXNIP induces the internalization of the glucose transporter GLUT1 and downregulates its transcription [113,117]. TXNIP overexpression in skeletal muscle cells reduces membrane GLUT1 expression, glucose uptake, and increases peripheric insulin resistance [118]. TXNIP deletion in murine embryonic fibroblast cells increases the levels of GLUT1 and the use of glucose by these same cells, and also increases lactate production [33,119]. Another study showed similar results with the GLUT4 transporter [120]. TXNIP is also overexpressed in the context of diabetes. Data from the literature identify TXNIP as a potential target in diabetes complications such as diabetic retinopathy, nephropathy, cardiomyopathy, and impaired post-ischemic revascularization [59,67,79,154,155,156,157,158,159,160,161,162,163,164,165,166,167,168,169,170,171,172,173,174,175,176]. In a rat model of diabetic cardiomyopathy, TXNIP deletion in cardiomyocytes induces an improved inotropic response to β-adrenergic stimulation [144].

Finally, in mice, glucose intolerance induced by High Protein High Fat Low Carbohydrate diet is also associated with an increase in TXNIP levels in the aorta [13,52]. In fact, high glucose and high fat levels are important inducers of higher endothelial TXNIP expression [99,177]. As reviewed elsewhere, glucose-induced higher tissue TXNIP expression has become a relevant therapeutic target not only to improve insulin secretion and sensitivity, but also for ameliorating the long-term microvascular and macrovascular complications of diabetes [67,145,178,179,180,181]. As the first piece of evidence, commonly used antidiabetic therapies are associated with a decreased expression of TXNIP [91,182,183,184,185,186], in particular via ChREBP and FOXO1 inactivation [121]. Indeed, endothelial dysfunction induced by high levels of TXNIP may have profound effects on the vasculature, a characteristic feature of metabolic disorders [13,20].

### 2.6. TXNIP is a Target of MiRNA

MiRNAs are also involved in glucose-induced TXNIP regulation. Analysis of the gene of TXNIP reveals several potential miRNA binding sites. [187]. For example, miR-17 downregulation by high glucose stabilizes TXNIP and removes TRX inhibition on ASK, leading to apoptosis [122]. Moreover, miR-33/TXNIP is believed to be essential in cell adaptation to bioenergetic demands [129]. Additionally, in numerous cardiovascular diseases, TXNIP has recently been identified as a target of miRNAs. For example, studies demonstrated a regulatory effect of miRNAs on TXNIP, resulting in oxidative stress control. In fact, by downregulating TXNIP, miR-370 or miR-20a protect endothelial cells from induced ox-LDL [126,131], and miR-20b protects endothelial cells from senescence. The relationship between miR-146a and TXNIP is involved in enhanced ROS production and vascular smooth muscle cell calcification [130]. TXNIP is also a regulatory target of miRNAs in pyroptosis. While an axis miR-497/TXNIP has been described in diabetic nephropathy, other studies report the role of different miRNAs, including miR-17 in the TXNIP/NLRP3 signaling pathway in inflammation-induced kidney injury or brain ischemia [123,124,125,128,132].

## 3. TXNIP is a Novel Marker in Cardiovascular Diseases

**Figure 2:**
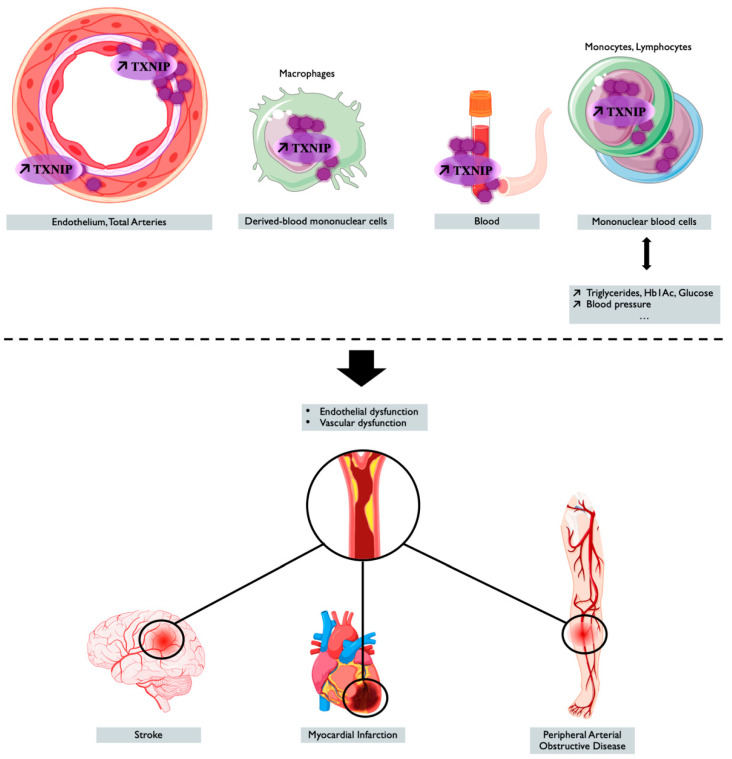
TXNIP overexpression is associated with cardiovascular outcomes and diseases. Tissue, blood levels of TXNIP, and the genetic regulation of TXNIP make it a potential marker associated with cardiovascular risk factors or cardiovascular event or diseases. Created with BioRender.com.

TXNIP is a genetic, blood, peripheral blood cells, and tissue ischemia marker associated with cardiovascular diseases, as summarized in Figure 2 and in Table 2, thus making TXNIP an interesting target for prognostic and treatment.

### 3.1. TXNIP as a Genetic Marker

#### 3.1.1. Genetic Variants of TXNIP

Even though a recent genetic study in a family with homozygous nonsense mutations shows that the suppression of TXNIP expression is non-lethal in humans, functional variants are reportedly associated with disease in the literature. Two different genetic variants of TXNIP can be described as genetic markers for cardiovascular risk. First, TXNIP rs7211 and rs7212 variants were significantly associated with glucose and blood-pressure-related phenotypes in the Brazilian general population, and TXNIP rs7211 was linked to arterial stiffness [24]. A more recent study reports that the rs7211 variant of TXNIP is a protective factor against obesity in non-diabetic subjects and in women in Mexican patients [151]. However, TXNIP rs7211 has not been found to be associated with retinopathy or with diabetes in Caucasian patients with type 2 diabetes (T2D) [233]. Interestingly, the same variants of TXNIP rs7212 and rs7211 are significantly associated with increased coronary artery disease risk, and the cumulative effects of these two SNPs have been described on coronary artery disease risk and the severity of coronary atherosclerosis in a Chinese population [26]. Finally, in this population, coronary artery disease risk is also found associated with the TXNIP DNA methylation level independently of TXNIP rs7211 and 7212 variants [26].

#### 3.1.2. Epigenetic Regulation of TXNIP

In addition to changes in the genomic sequence of TXNIP, epigenetic modifications, mainly influenced by environmental and lifestyle exposures, are also believed to contribute to cardiovascular disease risk. For instance, epigenetic regulations of the TXNIP gene via hyperglycemia have been described [188,189,234]. The most common epigenetic modification is DNA methylation, mainly found at cytosine-guanine dinucleotides sites within promoter regions and generally associated with gene silencing. The main DNA methylation described in the literature for the TXNIP gene locus is cg19693031, and this site is reported in numerous studies, in different tissues and in populations of different ethnic origin [27,235]. One of the 13 sites of methylation associated with blood pressure is cg19693031 at the TXNIP gene locus. In this study, decreased DNA methylation of TXNIP cg19693031 is related to increased blood pressure [28]. TXNIP methylation cg19693031 is also associated with lipid traits, and in particular with triglycerides levels [27,198]. In this two-stage epigenome-wide association study (EWAS), low TXNIP cg19693031 methylation is associated with high triglycerides levels independently of diabetes [27] in contrast to a previous study reporting an association of hypertriglyceridemia and genetic variation of TXNIP in diabetes patients [190]. In a Spanish EWAS, TXNIP cg19693031 is associated with prevalent T2D with TXNIP methylation inversely correlated with HbA1c levels in T2D [191], confirming the results obtained in the ESTHER cohort study [192], or more recently, in sub-Saharan African individuals with T2D [193]. The largest longitudinal study investigating DNA methylation in association with future risk of T2D in a multiethnic cohort reports that risk of future T2D was decreased per 1% increase in methylation at TXNIP cg19693031 site [RR = 0.92, 95%CI = 0.90–0.94]. Interestingly, this association surpasses further adjustment for non-genetic established risk factors for T2D [194]. A more recent study has been conducted in the British population to investigate the role of methylation in the etiology of T2D by investigating up to 11 years before T2D onset [195]. This study confirmed cg19693031 TXNIP methylation as a strong and consistent association with incident and prevalent T2D [195,196,197]. In fact, most epidemiological studies focus on T2D, probably related to the important role of TXNIP in glucose regulation by directly suppressing glucose uptake through binding to the glucose transporter Glut 1 [196,236,237,238,239,240]. In conclusion, the regulation of cg19693031 TXNIP methylation has been associated with cardiovascular diseases. However, today it is unknown whether cg19693031 TXNIP methylation plays a causal role in the development of diseases, remains a consequence of disease status, or is due to residual confounding. On the other hand, it will be important to consider the fact that the DNA methylation site is reversible, and therefore, aberrant DNA methylation modifications should in the future generate increased interest as drug targets.

### 3.2. TXNIP as a Blood Marker

Plasma levels of TXNIP may serve as a useful predictor of cardiovascular diseases. For example, carotid artery intima-media thickness is used as an indicator of atherosclerosis in patients with early-stage diabetes, and impaired glucose tolerance and is associated with increased plasma levels of TXNIP [30]. As a mechanical explanation, some works show, for example, that TXNIP overexpression induces endothelial dysfunction, vasoregulation disorders, and aortic stiffening via reduced levels of phosphorylated eNOS or NO bioavailability [12,21]. Moreover, TXNIP expression robustly correlates with the level of ROS production [241]. A study in diabetic rats also shows a correlation between circulating levels of TXNIP and the observation of aortic endothelial dysfunction with decreased levels of NO and VEGF and increased levels of ROS and VCAM-1 [17]. In contrast, lowering TXNIP blood levels by treating diabetic rats is associated with decreased aortic TXNIP levels, NO bioavailability, and arterial function [19]. In this study, TXNIP is also overexpressed at the aortic level, whether at the level of endothelial cells or VSMCs. Blood TXNIP levels are also found to be higher in stroke patients than in healthy controls [199]. Moreover, circulating TXNIP levels are also found to be increased after a heart attack induced by irradiation with 8Gy in rodents [55] and is associated with reduced TRX and TRX reductase levels, with increased cardiac TXNIP content and decreased cardiac antioxidant enzymes expression. In the literature, researchers are increasingly demonstrating a relationship between TXNIP blood levels and various bad outcomes beyond the cardiovascular field. For example, there is a correlation between TXNIP and peripheral nerve conduction velocity in patients with diabetes [242].

### 3.3. TXNIP as a Marker in Peripheral Blood Cells or Derived-Blood Cells

TXNIP levels can be altered in circulating blood cells, and it could be the signature of a risk factor for cardiovascular diseases. The expression of TXNIP is increased in the peripheral blood mononuclear cells of individuals with type 2 or type 1 diabetes and is correlated with the increase of inflammatory markers or endoplasmic reticulum stress [31,243,244]. TXNIP expression in peripheral blood mononuclear cells is increased in at-risk Takayasu arteritis patients. Interestingly, this TXNIP expression in peripheral blood mononuclear cells is associated with higher TXNIP expression in the aortic walls of these patients [200]. More specifically, TXNIP mRNA levels in leucocytes have also been investigated and are increased in patients with unstable angina pectoris [29] or with acute myocardial infarction [204], suggesting the role of TXNIP in atherosclerosis and the pathogenesis of cardiovascular diseases. Even if the endothelial levels of TXNIP are correlated with the adhesion of leucocytes, TXNIP levels in leucocytes also have an impact on the physiopathology of atherosclerosis [94,201]. For instance, TXNIP is one of the most significantly enriched genes in a subtype of macrophages resident in mouse atherosclerotic aortas [202]. In fact, TXNIP ablation has an atheroprotective effect through its regulation in the oxidative inflammatory response and atherosclerotic lesion development via a reduction in macrophage adhesion to VSMCs [110]. In addition, the deletion of TXNIP in leucocytes is reported to reduce leukostasis [80]. In vitro studies indicate that the mRNA expressions of NLRP3, IL-1, and IL-18 are up-regulated and positively correlated with the increased TXNIP mRNA in the peripheral blood leucocytes of coronary artery disease patients or THP-1 cells [203], which is consistent with the fact that TXNIP can promote vascular inflammatory responses and accelerate the process of atherosclerosis by activating the NLRP3 inflammasome [18,48,111]. TXNIP promotes inflammation and the activation of the monocytes in association with DNA demethylation, which orientates monocytes towards an inflammatory status through the NLRP3 inflammasome pathway [203]. Alternatively, one team tried to correlate TXNIP platelet content with platelet reactivity, atrial fibrillation, left atrial wall deformation, or tachycardia, but was not able to show any correlation [245,246,247,248]. Finally, OGA N-acetyl-glucosaminidase, which is the enzyme implicated in posttranslational modification of diabetic complications, has its mRNA levels correlated with TXNIP mRNA levels in the leukocytes of diabetic patients [205].

In the bone marrow, TXNIP could also have an impact in the mobilization of cells useful for post-injury or post-ischemic repair. TXNIP appears to be essential for maintaining the quiescence of hematopoietic stem cells and inhibits their mobilization. In fact, in the bone marrow, TXNIP decreases the Wnt signaling pathway and increases the interactions between hematopoietic stem cells and the niche [207]. In this study, the deletion of TXNIP in a transgenic mouse model promotes the proliferation of hematopoietic stem cells and their mobilization. Thus, TXNIP could inhibit the migration, differentiation, and mobilization of bone marrow cells via its antioxidant properties, but also inhibit the recruitment of these same cells to the ischemic site. Indeed, a more recent study from the same team suggests that TXNIP-p38 axis acts as a regulator mechanism in hematopoietic stem cell ageing; in particular, TXNIP increase the cell engraftment [208]. Additionally, a single-cell transcriptomic survey of aortas and coronary arteries in young and old primates reported, in the same way, FOXO3A loss (a transcription factor essential regulator of a pool of bone marrow cells and of oxidative stress level) as a key driver for arterial endothelial aging associated with the downregulation of TXNIP in smooth muscle cells [206]. However, the results have to be confirmed because the role of TXNIP in the mobilization of bone marrow cells is still poorly explored.

### 3.4. TXNIP as a Marker in the Context of Tissue Ischemia

The gene encoding TXNIP is induced by hypoxia in several cell types [249,250,251,252,253]. However, another group suggests that hypoxia induces a rapid decrease in the expression of mRNAs and proteins encoding TXNIP in an in vitro study [254]. The expression of TXNIP would therefore be regulated in a biphasic manner by hypoxia. First, TXNIP expression is rapidly reduced, and then its expression is increased under prolonged hypoxia. Based on these results, it appears that the expression of TXNIP can be upregulated or downregulated by ischemia, depending on the cell type and the pathological context. In vivo, ischemia is usually known to increase TXNIP levels [61,215,219,255,256,257]. Finally, the high sensitivity of TXNIP expression to a number of different stimuli suggests that TXNIP is a molecular switch that responds to various cellular stresses and regulates several molecular mechanisms in ischemic injuries such as oxidative stress and inflammation [253,258]. Moreover, knowing TXNIP involvement in ROS production and inflammation, which leads to endothelial dysfunction, TXNIP may also be implicated in vessel damage. Then, these vessel alterations can culminate in the occurrence of ischemic diseases.

#### 3.4.1. TXNIP as a Marker in Myocardial Ischemia

In a study on cardiac ischemia-reperfusion, the authors showed that the NLRP3 inflammasome is increased in cardiac endothelial cells via TXNIP [209]. Although the rapid restoration of coronary flow is essential for the rescue of heart muscle, reperfusion is inevitably accompanied by sterile inflammation, which has been widely studied to be the primary cause of myocardial damage and dysfunction. Indeed, this inflammation can lead to ventricular remodeling and heart failure [210,211]. The increased activation of the inflammasome is evidenced by increased expression of NLRP3 and caspase-1 activity followed by the increased production of IL-1β and IL-18. The intramyocardial injection of anti-NLRP3 siRNA or the intraperitoneal injection of an inflammasome inhibitor results in an attenuated infiltration of macrophages and neutrophils and a decrease in ischemia-reperfusion damage as measured by apoptosis of cardiomyocytes and the size of the infarct. The intramyocardial injection of anti-TXNIP siRNA also decreases the size of the infarction and the activation of NLRP3, which suggests the value of targeting TXNIP to prevent deleterious effects of ischemia [209]. As a matter of fact, increased endothelial expression of TXNIP was found in diabetic hearts, which correlated well with the fact that insufficient angiogenesis aggravated cardiac remodeling and caused poor survival following myocardial infarction [173]. These results are in agreement with a previous study using intramyocardial injection of anti-TXNIP siRNA in the context of diabetes, which reduces oxidative stress, apoptosis and ischemia-induced myocardial damage [216]. A recent study shows that the interaction between TXNIP and NLRP3 is the key point in the damage-induced myocardial ischemia. Preventing the interaction between TXNIP and NLRP3 suppresses the ROS-TXNIP-NLRP3 pathway and alleviates myocardial ischemia/reperfusion injury [95,259]. In recent years, a growing body of research has begun to target TXNIP and thus suppress the ROS-TXNIP-NLRP3 pathway to hamper heart damage in myocardial ischemia [109]. A plethora of inhibitors proposed to hamper post-myocardial ischemia damage inhibit hypoxia-induced TXNIP and NLRP3 expressions [257]. This strategy is believed to improve cardiac function and reduce atrial fibrillation after myocardial infarction [100]. In addition, the administration of a vector-encoding TRX in diabetic rats increases capillary and arteriolar density, and therefore improves the restoration of cardiac function after myocardial infarction in the context of diabetes [260]. The balance of the TRX-TXNIP system is essential for the survival of cardiomyocytes in the context of ischemia [261]. In ischemic cardiomyopathy, the TRX-TXNIP system is impaired with reduced TRX and overexpressed TXNIP, whereas these features are not observed in dilatated cardiomyopathy [262]. TXNIP upregulation and the subsequently increased formation of the TRX-TXNIP complex is a proposed pathway by which diabetes induces insufficient angiogenesis and thereby exacerbates myocardial ischemia injury [215]. Moreover, in rats, treatment with resveratrol, a well-known inhibitor of TXNIP, is reported to be cardioprotective and to promote revascularization in a model of myocardial infarction. In this study, the authors show that resveratrol induces an increase in the expression of TRX and VEGF in a dose-dependent manner [263]. Again, novel strategies or several compounds proposed as treatments for myocardial infarction are regulators of the TRX-TXNIP system [64,264,265,266,267]. In addition to inflammation and dysregulation of the oxidative state trough the TRX-TXNIP balance, autophagy is also a mechanism where TXNIP plays a critical role in myocardial injury via the TXNIP/Redd1 pathway [64]. The heart responds to oxygen deprivation by increasing glucose uptake and glycolysis. Given the crucial role of glucose supply in the cardiac response to ischemia and the role of TXNIP in glucose uptake via GLUT1, it is likely that the resulting increase in glucose supply is due to TXNIP deficiency, and it provides cardioprotection to the ischemic heart. Indeed, the suppression of TXNIP in cardiomyocytes in mice was found to confer a protective advantage on the ischemic heart [212] as well as on left ventricular hypertrophy and heart failure [213,214].

#### 3.4.2. TXNIP as a Marker in Hind Limb Ischemia

The role of the TRX-TXNIP system in the occurrence of pathologies such as arteriopathy of the lower limbs, and therefore ischemia of the lower limbs, has been poorly studied. Transgenic mice overexpressing endothelial TRX promote angiogenesis and post-ischemic arteriogenesis [268]. Indeed, in this study, the overexpression of TRX improves endothelial function via a decrease in ROS and an increase in the NO bioavailability. This study is interested in mitochondrial TRX, but an improvement in post-ischemic revascularization in the same model of lower limb ischemia is also observed with endothelial overexpression of cytosolic TRX [217]. The role of TXNIP, using paw-level anti-TXNIP siRNA injections in a mouse model of lower limb ischemia and diabetes, provides some insight. The team shows that the angiogenesis defect attributed to diabetes is dependent on TXNIP. Indeed, targeting TXNIP helps to counter the deleterious effects of diabetes through improved reperfusion of the ischemic limb, reduction of tissue damage, and increased capillary density [154]. In fact, the inhibition of TXNIP expression using fenofibrate treatment also helps to counter the post-ischemic revascularization defect of the lower limbs associated with diabetes [218]. Thanks to a transgenic model, TXNIP deletion is also involved in reducing the deleterious effects of a fatty diet in the revascularization of the lower limbs after ischemia [269]. Moreover, targeting specifically endothelial TXNIP protects from metabolic-disorder-related impairment in post-ischemic revascularization and tissue recovery [21].

#### 3.4.3. TXNIP as a Marker in Cerebral Ischemia

TXNIP is also of growing interest to those who study neurological diseases, including cerebral ischemia, as previously reviewed [270]. In cerebral ischemia, four major pathologies are described: ischemic stroke, subarachnoid hemorrhage, neonatal hypoxic-ischemia, and vascular dementia. A number of studies have investigated the role of the TRX-TXNIP system in cerebral ischemia. In fact, a high serum TRX level is a good prognostic marker in ischemic stroke [271]. Cerebral ischemia induces activation of the inflammasome and is characterized by an increase in NLRP3 and TXNIP. When TXNIP and NLRP3 are decreased, tissue damage associated with cerebral ischemia is reduced [219,220]. Many other original compounds with antioxidant properties are able to decrease the expression of TXNIP and attenuate brain damage and neurotoxicity following ischemia by suppressing the activation of the inflammasome [103,127,221,222]. Nrf2 is reported to inhibit the NLRP3 inflammasome by regulating the TRX/TXNIP complex and consequently hampers cerebral ischemia reperfusion injury [102]. Increasing the expression of Nrf2 subsequently decreases the expression of TXNIP, NLRP3, Cleaved Caspase-1, and IL-1β, and reduces the infarction volume and improved neurological outcomes after middle cerebral artery occlusion [60]. Other authors support the idea that TXNIP silencing alleviates oxidative stress injury by regulating the MAPK-Nrf2 axis in ischemic stroke [61]. In addition to regulating Nrf2 expression with different compounds, a critical mechanism associated with the downregulation of TXNIP is the nuclear translocation of Nrf2 promoted by AMPK and GSK-3β [108]. This signaling pathway is in line with the improvement of the oxidative defense with augmentation of TRX and the diminution of TXNIP expression [225]. TXNIP upregulation is associated with blood–brain barrier (BBB) disruption in response to experimental hyperglycemic stroke with an increase of BBB permeability trough the TXNIP / NLRP3 inflammasome axis [223]. In a metabolic stress context, TXNIP is also upregulated and associated with the loss of mural cells since targeting TXNIP with different strategies hampered the signalization cascade of the NRLP3 inflammasome and induced protection against the loss of cells [13,21]. Moreover, TXNIP expression increases in the cytoplasm of neurons with significant brain damage due to focal cerebral ischemia in mice. The inhibition of TXNIP using anti-TXNIP siRNA protects neuronal cells and increases cell viability [224]. TXNIP, with its pro-inflammatory and pro-apoptotic effects, widely participates in early brain injury after subarachnoid hemorrhage [228,229]. In addition, NADPH oxidase-dependent inflammasome activation appears to contribute to traumatic brain injury pathology via a mechanism associated with TXNIP [58]. Again, the overexpression of TXNIP is linked to inflammasome activation or reticulum endoplasmic stress [62,230]. TXNIP is also involved in neonatal hypoxic-ischemia, which occurs in the youngest neonates. In rat models of the disease, PPAR-β/δ agonist mitigates apoptosis and reduces NLRP3-related neuroinflammation by increasing the miR-17-5p level and decreasing TXNIP expression [124,125]. The PPAR-β/δ/miR-17/TXNIP pathway is able to control TXNIP expression and subsequently inhibits NLRP3 activation [231]. Moreover, the ROS/TXNIP/NLRP3 pathway plays an important role in hemorrhagic transformation [226]. Finally, targeting TXNIP seems to also be an interesting target in vascular dementia since acupuncture shows neuroprotective effects by decreasing TXNIP and NLRP3 expression-associated oxidative stress and inflammation in a rat model of the disease [232]. These neuroprotective properties have also been reported in ischemic stroke [227].

## 4. Conclusions

Although larger-scale clinical studies need to be performed to use TXNIP as a biomarker in the clinic, TXNIP appears to be a preferred therapeutic target in endothelial and vascular dysfunction to prevent cardiovascular complications associated with age, metabolic disorders, and oxidative stress-related disorders. Indeed, the overexpression TXNIP is now well-recognized as being deleterious and an emergent new suspect for cardiovascular risk and diseases, as summarized in the diagram (Figure 2). Moreover, the action of drugs used in the treatment and prevention of cardiovascular pathologies with effects on the expression of TXNIP indirectly suggests the therapeutic benefit of targeting TXNIP. Therapies targeting endothelial TXNIP could thus delay endothelial dysfunction and the onset of cardiovascular complications induced by aging and its comorbidities.

## Figures and Tables

**Table 1:** The multiple signaling partners of TXNIP and pleiotropic effects.

Function	Signalling Partner	References
Shuttle	TRX	[36,37]
	HIF1a	[38]
	NfkB	[39,40]
	Itch	[41,42,43]
Prooxydant	TRX	[13,21,32,40,44,45,46,47,48,49,50,51,52,53,54,55]
	NADPH oxidase	[13,21,51,52,56,57,58]
	AMPK/NrF2	[59,60,61,62]
	Redd1	[63,64]
Proinflammatory	NLRP3	[13,18,21,60,65,66,67,68,69,70,71,72,73,74,75,76,77,78,79,80,81,82,83,84,85,86,87,88,89,90,91,92,93,94,95,96,97,98,99,100,101,102,103,104,105]
	AMPK/NrF2	[60,62,90,92,102,103,104,106,107,108]
	NF-κB	[39,40,72,83,109,110]
	Kruppel-like factor 2	[111]
Metabolism	AMPK	[92,112,113]
	MondoA	[114,115]
	IGF1	[116]
	Glut1	[33,113,117,118,119]
	Glut4	[120]
	ChREBP/FOXO1	[121]
Target of miRNA	miR-17, miR-17-5p	[122,123,124,125]
	miR-20a, miR-20b	[126]
	miR-25-5p	[127]
	miR-30c-5p	[128]
	miR-33	[129]
	miR-146a	[130]
	miR-370	[131]
	miR-497	[132]

**Table 2:** TXNIP as a marker of cardiovascular risk and disease.

	Location	Parameter or Disease	References
Genetic Marker	TXNIP rs7211 variant	Arterial stiffness, obesity	[24,151]
	TXNIP rs7211- rs7212 variants	Glucose, blood pressure, coronary atherosclerosis	[24,26]
	Various epigenetic changes	T2D	[188,189]
	DNA methylation cg19693031	Blood pressure, T2D, coronary artery disease	[26,27,28,190,191,192,193,194,195,196,197]
		Triglycerides and/or HbA1C levels	[27,190,191,192,193,198]
Blood Marker	Plasma or serum levels of TXNIP	Carotid Intima Media Thickness	[30]
		Stroke or heart attack	[55,199]
		Diabetes associated macrovascular endothelial dysfunction	[17]
		Diabetes associated vascular complications	[19]
mRNA Marker	TXNIP in peripheral blood cells	At-risk Takayasu arteritis, atherosclerosis, coronary artery disease, leukostasis	[80,94,110,200,201,202,203]
		Unstable angina pectoris, acute myocardial infarction	[29,204]
		Diabetes associated macrovascular endothelial dysfunction	[17]
		Diabetes associated vascular complications	[19,205]
	TXNIP in cardiac tissue	Heart attack	[55]
	TXNIP in aortic tissue	At-risk Takayasu arteritis, atherosclerosis, arterial aging	[51,200,202,206]
		Diabetes associated macrovascular endothelial dysfunction	[17]
		Diabetes associated vascular complications	[19]
Tissue Marker	TXNIP in bonne marrow	Mobilization of cells	[202]
	TXNIP in Myocardiac ischemia	I/R damage (infarct size or ventricular remodeling or heart failure or atrial fibrillation)	[207,208]
		I/R damage in diabetic hearts or survival	[64,100,109,209,210,211,212,213,214]
	TXNIP in Hind limb ischemia	Reperfusion of ischemic limb, tissue-recovery, capillary density in diabetic mouse	[173,215,216]
		Reperfusion of ischemic limb, tissue-recovery, capillary density in mouse with fat diet	[217,218]
	TXNIP in cerebral ischemia	Ischemic stroke	[58,60,61,102,103,108,127,219,220,221,222,223,224,225,226,227]
		Subarachnoid haemorrhage	[62,228,229,230]
		Neonatal hypoxic-ischemia	[124,125,231]
		Vascular dementia	[232]
